# India Biodiversity Portal: An integrated, interactive and participatory biodiversity informatics platform

**DOI:** 10.3897/BDJ.4.e10279

**Published:** 2016-11-07

**Authors:** Thomas Vattakaven, Rohit M George, Dhandapani Balasubramanian, Maxime Réjou-Méchain, Gowrappan Muthusankar, Brahmasamudra Ranganna Ramesh, R Prabhakar

**Affiliations:** ‡ Strand Life Sciences, Bangalore, India; § French Institute of Pondicherry, Puducherry, India; | Ashoka Trust for Research in Ecology and the Environment, Gangtok, India; ¶ UMR AMAP, IRD, Montpellier, France

**Keywords:** Biodiversity informatics, Biodiversity, Biodiversity data, Species distribution, Species information, Citizen science, Crowd-sourcing, Data, Information te﻿chnology, Open data, Conservation, Public participation in scientific research, India

## Abstract

**Background:**

This paper describes a growing biodiversity platform, launched in 2008, which organizes knowledge on the biodiversity of India. The main objective and originality of the India Biodiversity Portal (IBP) is to aggregate curated biodiversity data of different kinds (e.g. distribution maps, temporal distribution or life history) in an integrated platform where amateurs and experts can easily interact.

**New information:**

Since its launch, the platform has seen an exceptional increase in both user activity and biodiversity data. Currently the portal has descriptions of over 20,400 species, and has aggregated approximately 1,280,000 observations covering more than 30,000 species, which already constitutes a unique source of information for scientists and stakeholders in conservation. Over 8500 users have registered on the portal. The amount of data generated and to be generated in the next few years by this portal will certainly help the effective implementation of biodiversity conservation and management in one of the most ecologically diverse countries in the world.

## Introduction

In many areas of the world, much remains to be done to understand biodiversity dynamics in space and time (Reid and Miller 1989). This is especially true in the tropics, where ecosystems are still experiencing major changes in their structure and composition due to global changes (Malhi et al. 2014). At least three major knowledge gaps can be depicted: (i) the lack of knowledge on species distribution, an impediment termed the Wallacean shortfall, which limits considerably our ability to support conservation and management plans in a changing world; (ii) the lack of knowledge on taxonomy/phylogeny, named the Linnean shortfall , which is a major barrier to effective biodiversity monitoring strategies and; (iii) the lack of knowledge on species ecology and behavior, (Eltonian and Raunkiaeran shortfalls), which limits our ability to predict the effects of global changes on natural populations and ecosystems (Hortal et al. 2015). Other shortfalls in biodiversity knowledge listed by Hortal et al. 2015 include knowledge gaps in our understanding of species evolution, population abundance and responses to abiotic conditions.

Most of the knowledge produced on biodiversity remains fragmented and is not available in any standardized structure (Boakes et al. 2010, Amano et al. 2016,Ellwood et al. 2015) impeding for instance, our ability to perform robust analyses on species distribution patterns (Whittaker et al. 2005). However, generating accurate data on biodiversity over large spatial and temporal scales is an extremely difficult challenge to meet without a multi-institution and/or multi-actor collaborative schemes. With the advent of open access biodiversity data aggregation initiatives, large-scale involvement of citizens in documenting biodiversity data, information technology advancements and an increasing willingness to share data, open data on biodiversity is significantly growing (Reichman et al. 2011, Silvertown 2009). For instance, the Global Biodiversity Information Facility (GBIF), currently hosts more than 650 million records of species occurrence and the Map of Life (Jetz et al. 2012), has over 370 million observations on nearly a million species. Many other citizen-science based (e.g. iNaturalist, iSpot, eBird) or collection-based [e.g. Integrated Digitized Biocollections (iDigBio), invertnet (Dietrich et al. 2012)] initiatives, have generated billions of records conforming to existing biodiversity informatics standards. Other initiatives such as the Encyclopedia of life (EoL) provide descriptive information on species, while still others such as the Biodiversity Heritage Library focus upon biodiversity literature and making it available digitally under open access. Most of these initiatives focus on a single aspect of biodiversity (e.g. species distribution, taxonomic knowledge or literature) and very few combine different kinds of biodiversity data and make it available under a species axis. One such initiative is the Atlas of Living Australia, which is experiencing great success as a biodiversity informatics platform. Such initiatives may serve as a good models for country wide or region based initiatives to collect, curate and disseminate biodiversity information.

India is one of ten largest countries in the world with a total area of more than 3 million km². India is also well-recognized as a highly ecologically diverse country, with a high diversity of habitat, ranging from alpine to tropical ecosystems, containing high level of endemicity (Ahmedullah and Nayar 1987, Ramesh and Pascal 1997) and one of the eight mega-diverse hotspots in the world (Mittermeier et al. 2011, Olson et al. 2001, Myers et al. 2000). Although it occupies only 2 percent of global space, India contains nearly 7 percent of global faunal diversity (Mathur et al. 2014). Though Indian biodiversity has been extensively and systematically studied, at least since the 16th century (Bhattacharyya 1982), data on species ecology and distribution is still hard to access and largely scattered among institutions or individuals without much interactions within and between taxonomists and ecologists. Although India has spawned several biodiversity aggregation initiatives, most of them have focused on specific objectives and have gained specific strengths. For instance, SeasonWatch and MigrantWatch are engaged in citizen science to gather data on seasonalities of trees and migration of birds respectively. The Indian Medicinal Plants Database documents information on medicinal uses of plants in Indian systems of medicine. Indian Bioresource Information Network and Indian Biodiversity Information System are known multi-institutional and institutional repositories hosting species information. However, a comprehensive biodiversity information system that can compile various aspects of biodiversity information at the species level, such as spatio-temporal distribution, taxonomic and conservation status, or any relevant information on ecology/ethology, is currently lacking. This gap may be filled by an inclusive model that allows broad based participation from experts and enthusiasts. Such an information system is thought to be crucial towards implementing India’s aims towards the conservation of its biological resources and associated knowledge and facilitating access to them in a sustainable manner (Gadgil 2003).

In this paper, we introduce the India Biodiversity Portal (IBP), an initiative building on a collaborative platform that integrates an array of biodiversity knowledge and serves it under open access. As discussed below, the core philosophy of this platform is to bring together expertise from research, civil society, advocacy, information technology and legal domains with a strong commitment to open data for effective biodiversity conservation in India.

## Project description

### Title

Overall description of IBP

### Design description

**History and Core Philosophy of IBP**

The India Biodiversity Portal is an open access biodiversity information platform for India launched in December 2008, involving various institutions in India (listed in the acknowledgements). IBP has two main objectives: aggregating curated biodiversity data for all species in India and creating/stimulating social networks where biodiversity amateurs and experts can interact. The portal is participatory and all information is freely and openly accessible by any member of the public under Creative Commons licences*^1^. IBP operates on the belief that biodiversity and conservation information are essential social goods that should be freely available for all to access.

**Modules and Technology**

**Figure 1.**
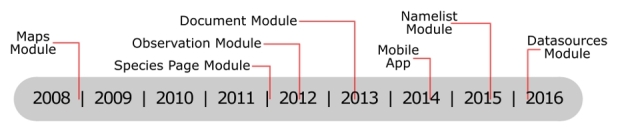
Timeline of the deployment of some of the main modules and features on the India Biodiversity Portal.

**Figure 2.**
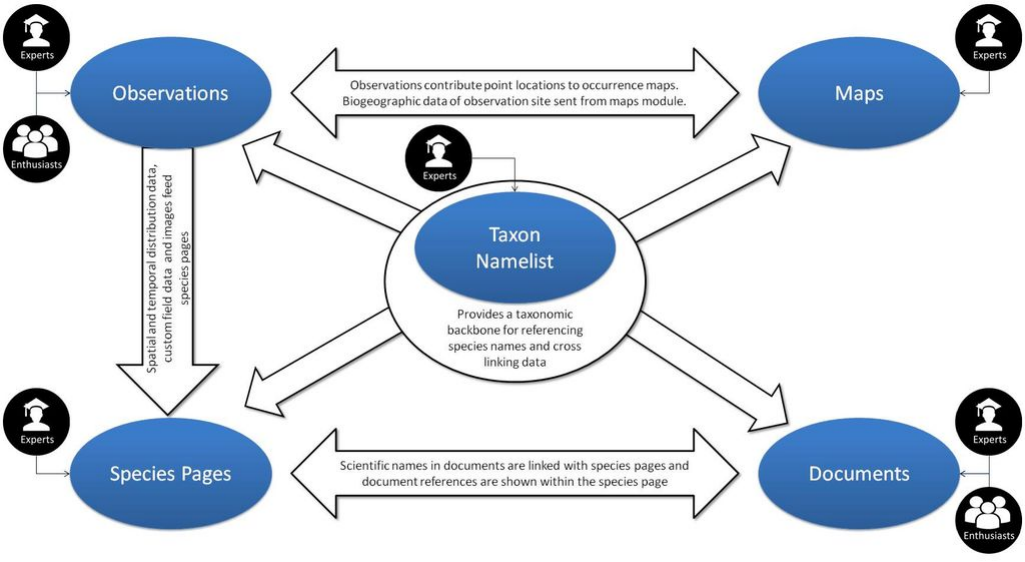
The relationships between different modules on the India Biodiversity Portal. Experts and enthusiasts collaborate in sourcing and validating information within each module.

The India Biodiversity Portal consists of several interconnected modules:

The “Maps module” was the first module to be completed and deployed on IBP (Fig. 1). Built on the open source GeoServer (Deoliveira 2008), all layers are categorized into thematic and geographic categories including biogeographical information, land use/land cover and administrative units. The majority of the layers are under Creative Commons licenses, freely sharable and downloadable by users. The “Maps module” is connected with the other modules on the portal (Fig. 2). Map layers can be overlaid and the attributes can be queried at any location. There is a generic search and a species search function on the map module.

The “Species Pages module” was deployed in 2011 with the objective of building and serving a descriptive page for every species occurring in India. As no comprehensive species-lists cataloguing all species occurring in India were available, species names of known distribution in India were aggregated from global biodiversity databases such as the GBIF, IUCN, Fishbase (Froese and Pauly 2016) and other regional data to create stub species pages. The structure of the Species Pages Module follows the international standards of the Taxonomic Database Working Group. An adaptation of the Species Profile Model and the Plinian Core format was used to set up the standard data fields for storing data within species pages. The structure has the flexibility to accept information on varied subjects such as taxonomy, natural history, habitat and distribution of species, and allows content contribution in every field from multiple contributors. Species information is populated from species contributors who are recognised experts in their respective taxonomic group, identified and validated by a team of administrators from IBP. The contributors can input information as primary contributions or source data from scientific literature, species assessment surveys and from content held in institutions. All information is input with appropriate attribution and references to the original sources. Experts are also able to validate and extract images from “observations” uploaded by the general public to enhance the content of the species pages. Some components of a species page such as the occurrence map and documents are dynamically updated as information is populated for the taxa in the respective modules of the portal. Species pages content can be exchanged with other initiatives and downloaded under the Darwin Core Archive format (Wieczorek et al. 2012).

**Figure 3.**
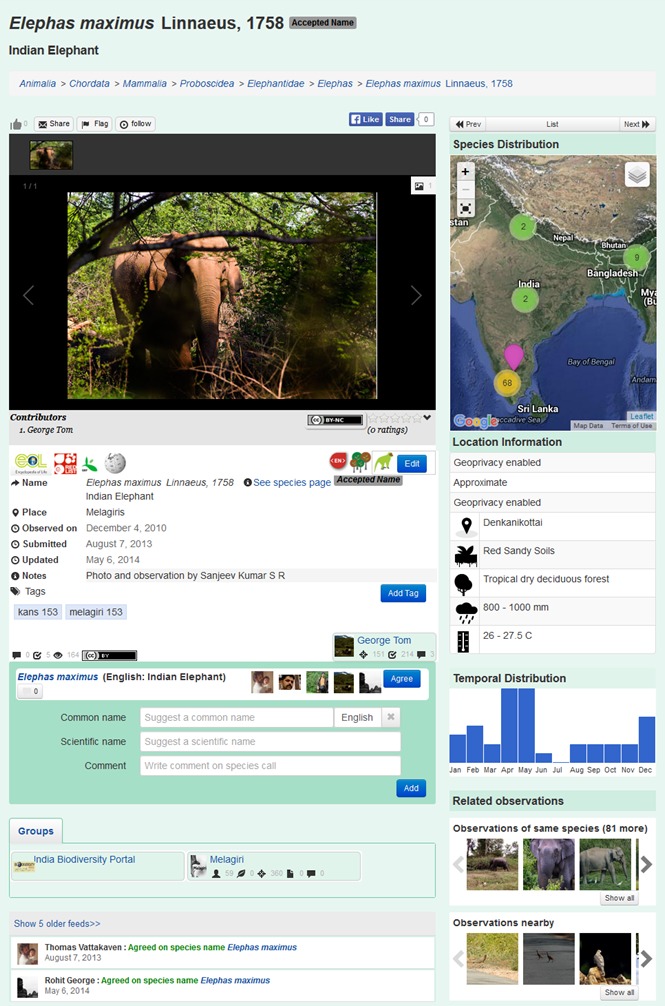
An observation page on the portal. In this example of an observation on IBP, the animal was observed by a user on the 4th of December 2010 and uploaded to IBP in 2014. The user added the scientific name *Elephas maximus* Linnaeus, 1758 and the common name “Indian Elephant”. Four other users agreed with his identification. The new observation is displayed as an occurrence point on the distribution map (top right) with information on site-specific environmental conditions and temporal distribution of all observations of that species.

The “Observation module” on IBP facilitates crowdsourcing of biodiversity information and participation through citizen science, to aggregate spatial and temporal species distribution data. It was deployed in 2012 and allows users to upload biodiversity observations with supporting media (image, audio or video), location, date and a taxonomic name (if known) along with observation notes (Fig. 3). Geoprivacy of the location can be enabled in observations for which the uploader wishes to obscure the precise location. Eg: for observations of endangered species. Interested participants can interact on an observation by helping identify the documented organism, agreeing with an existing identification and commenting upon the observation. Multiple participants may agree upon a suggested name and the maximum voted name is chosen by the system as the reigning name for the observation. Species curators may also validate a name to lock the observation identity. On identification and allotment of a scientific name, observations are automatically associated with the corresponding species page and location coordinates are instantly added to the occurrence map of the species. Furthermore, the date of its observance is harnessed for plotting its temporal distribution; custom fields may contribute to specific species page fields and images from the observation becomes available for experts to validate and be extracted into the species page gallery. The portal also has the ability to accept species checklists, where each row is treated as an individual observation, with an option to include media.

The “Documents module” was developed in mid 2013 to gather and harness the wealth of biodiversity information present in both academic journals and grey literature. Any registered user can contribute documents as PDF files along with metadata such as the location, date, attribution and description. The Portal harnesses the Global Names Recognition and Discovery service to run a procedure to automatically extract species names from uploaded documents and link these documents with the respective species pages.

The name curation or "Namelist module" allows taxonomists to account for all species names on the portal and organise them by updating the name with metadata such as the current taxonomic status, taxonomic classification, author and year of publication. Names can be resolved against global species databases such as the Catalogue of Life (CoL, Roskov et al. 2015), GBIF, EoL or by curator input. The provenance of the name status is maintained and displayed to track changes. It also facilitates taxon curators to segregate names into categorized lists based on the validity of a name with respect to geography and taxonomy allowing dynamic species lists for India to be generated and maintained. The name curation exercise is also crucial to building a unified management classification and a taxonomic tree that facilitates the organization and navigation of all species content throughout the portal.

Along with the primary modules, IBP also has a number of features and tools to help record, collect and curate data. The “Groups” feature, allows the creation of a micro-site on any theme of interest within IBP. Moderators may pull relevant elements from the main modules into the group. The groups may be taxon-based, location-based or based on some other theme of interest (e.g. "Medicinal Plants", "Roadkill Network" and "ButterflyIndia"). Group moderators have the ability to create "custom fields" for observations uploaded to the group, i.e. any query that might be relevant for species ecology (such as queries related to phenology, microhabitat or larval stage). These fields are displayed as queries in any observation added to the group. For instance, members from the "Spotting alien invasive species" group may add values to a custom field such as "Is the specimen recorded in the observation fruiting or flowering?”. The groups are a convenient way for users with an interest in a specific species group or habitat to build a network and interact upon thematic content or conduct citizen science campaigns on the theme of interest. They are currently 49 groups on the portal (as on 25 July, 2016).

A "Datasources module" has recently (2016) been deployed to facilitate aggregation and integration of observation data from external data sources such as GBIF with the portal. The module integrates these records by replicating spatial, temporal and species name attributes of the record on IBP and creating an observation. Media objects are not replicated but will be displayed within the observation gallery from its original source URL if available. This functionality provides IBP with powerful capabilities to aggregate spatial and temporal species records from regional and global databases to enhance its data.

A mobile application for Android and iOs smart phones has been developed for users with portable devices. These applications make it easier to record observations, since the date and location of the observation are recorded directly by the device. The Android app is available for download on the Google Play Store and has had over 2200 downloads (as on 25 July, 2016). The iOS version of this application has also recently been released through the iTunes app store.

**Content and participation**

As a part of its objective of aggregating a species list for India and creating a descriptive page for every species in the country, IBP has so far aggregated over 48,986 species names from global sources. Species pages have been created for these names and over 40% (20,208) have content populated in them through contributions by a diverse array of species experts. As depicted in Fig. 7, some taxonomic groups, such as angiosperms or birds, have more species pages than the currently estimated number of species, while some other groups, such as Fungi or Lichen, are poorly documented in IBP. Much effort will be needed, not just to compile complete lists of species in India, but also to resolve taxonomic synonymy with current taxonomic standing, classification and publication details. IBP’s taxon-name curation interface (Namelist module) was recently deployed to facilitate this task. Expert participation will help to track synonyms, correct misspelt names and peg infra-species names under species leading to more taxonomically accurate species lists which will serve as a taxonomic backbone for the portal.

Experts who wish to contribute species page content can request permissions online at any taxon level. A team of administrators verify the requester’s expertise in the concerned taxonomic group and allot permissions. Over 100 experts in different taxa have already registered with the portal and contributed content. Continued recruitment of experts as contributors and participation by registered experts through an online species create/edit system ensures that the portal will continue to aggregate expertise and content.

**Figure 4.**
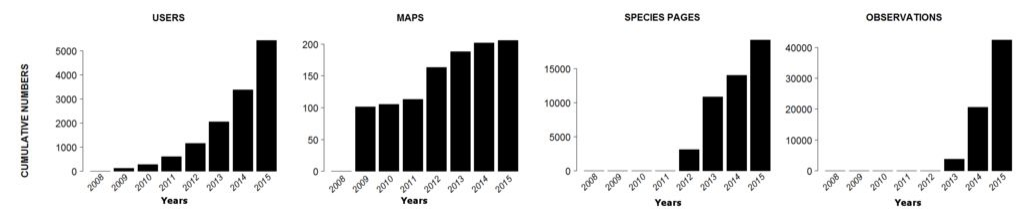
Temporal dimensions of data aggregation on the portal in its primary modules since its launch in 2008.

**Figure 5.**
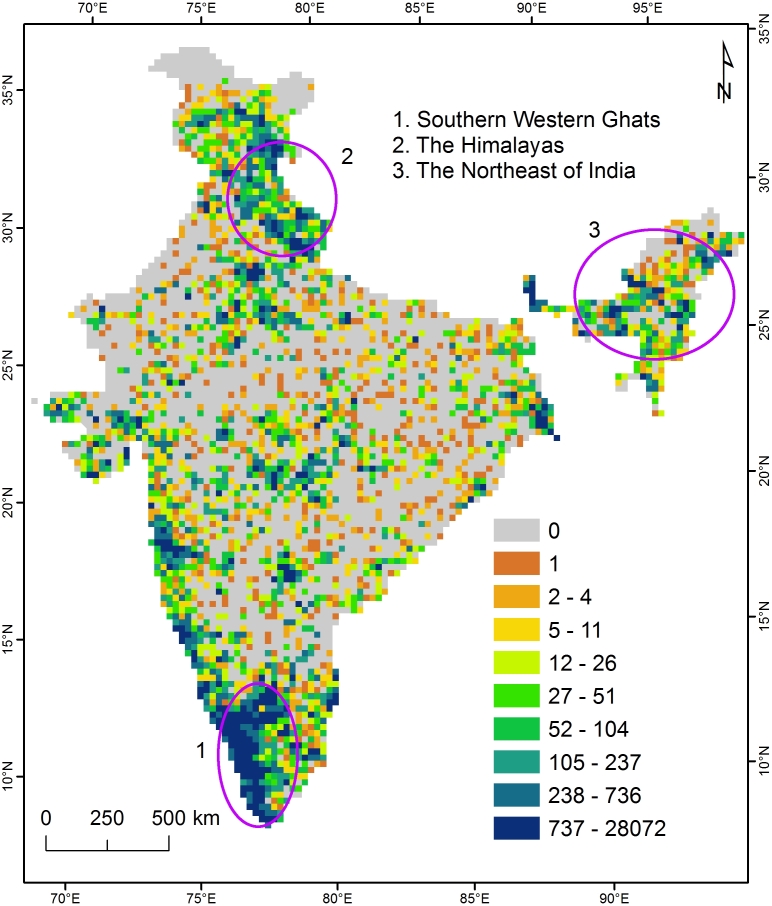
The spatial distribution of all observations on IBP at 25-km resolution, with locations of biodiversity hotspots indicated (all data as on 25/07/2016).

**Figure 7.**
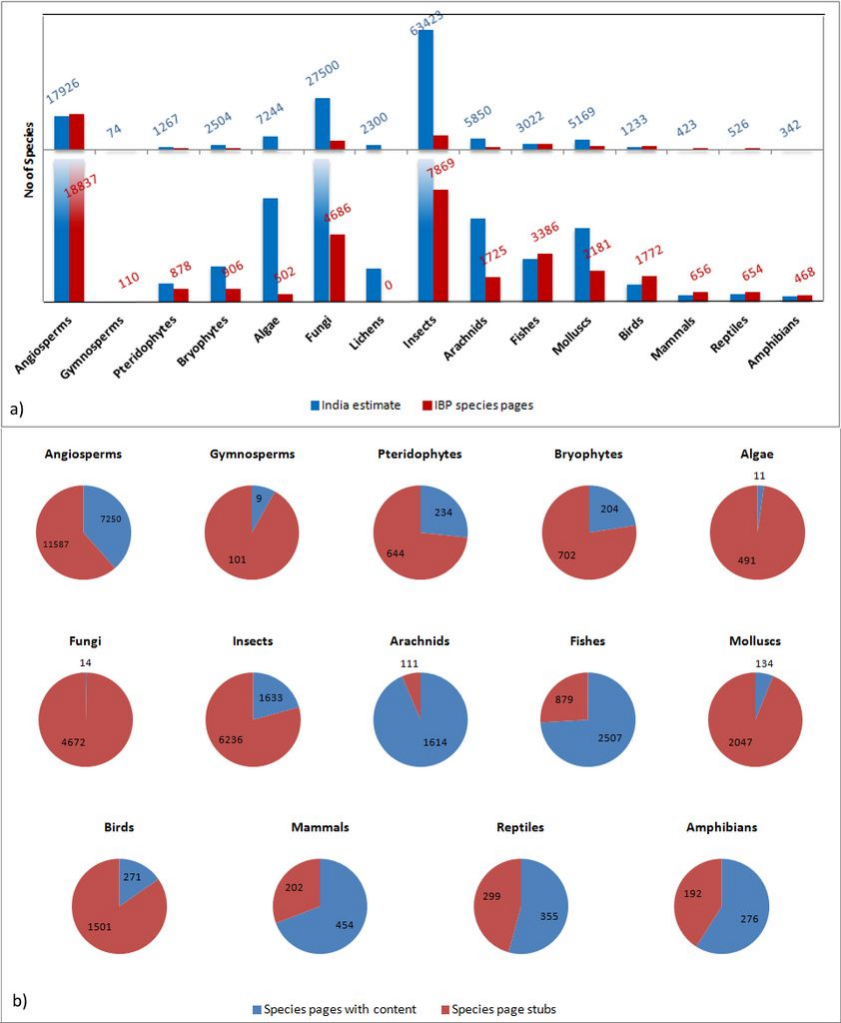
**(a)** Comparison of the numbers of species pages per taxa in the India biodiversity portal, with those reported in the India's fifth national report to the convention on biological diversity held by the Ministry of environment and forests, Government of India (Mathur et al. 2014). The bottom panel is a magnified view of the top panel. Cases where the IBP coverage exceeds the India estimate are partlly due to taxonomic variations caused by aggregation of data from different sources. **(b)** Pie charts showing the availability of content within species pages for various taxa groups on IBP. Species stubs are placeholder pages with skeletal content.

**Figure 8.**
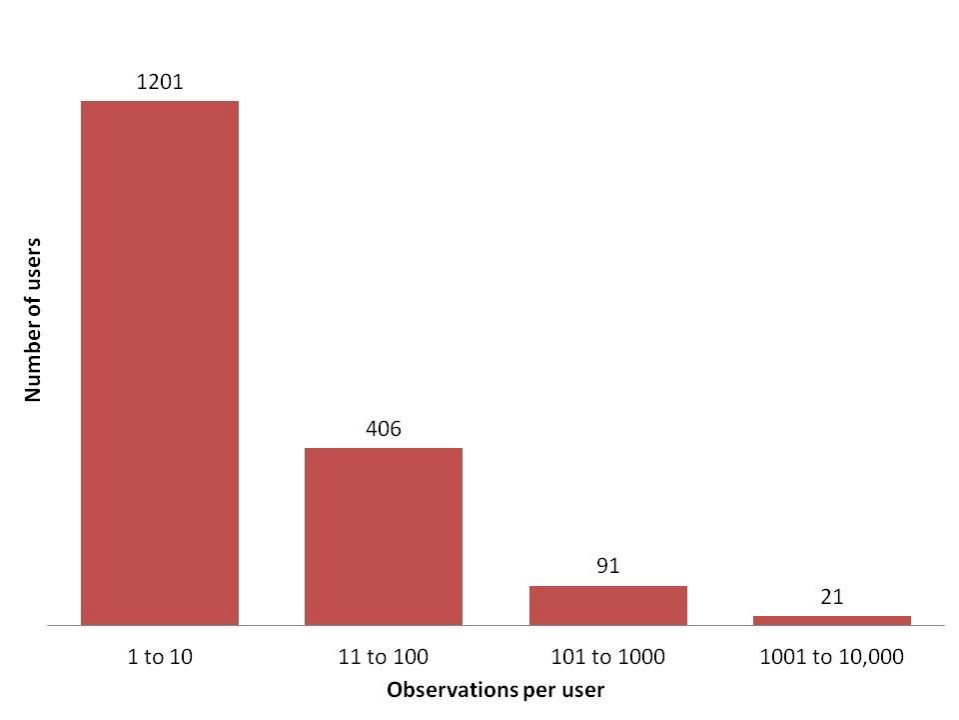
Over 20% of the 8500 registered users on IBP have uploaded at least one observation and more than 6% of the users have ten or more observations. "Super users" who have contributed over a thousand observations constitute over 0.2% of the users.

As illustrated in Fig. 4, IBP has seen almost exponential growth in the number of users with over 8500 registered users currently and growing at a rate of over 400 users per month. Statistics revealed by Google Analytics indicate that there are over 86000 page views and approximately 18,000 active visitors per month. Contribution in all of the modules have been on the rise, especially in the Observation Module, with over 4000 observations being uploaded every month. Over 20% of the registered users have uploaded observations on the portal with 15% contributing less than 10 observations per user and over 1% uploading more than 100 observations (Fig. 8). Given the current trends in participation, we expect that these figures will continue to grow exponentially in the future. So far, 132,070 observations have been aggregated through user participation and an additional 1,149,228 records from global databases aggregated through its "datasources module". These records contribute spatial data to the occurrence maps of over 15,000 species pages. From a spatial distribution perspective, there is high density of observations recorded from Southern Western Ghats, the Himalayas and the Northeast of India (Fig. 5), all of which comprise parts of biodiversity hotspots (Myers et al. 2000).

**Example case**

On April 2, 2014, a user (Sunny Joseph), uploaded an observation of an unidentified spider, feeding upon ants, on the observation module of IBP (Fig. 3A). The observation was tentatively identified as *Siler semiglaucus* (Simon, 1901) by a species expert (Siddharth Kulkarni). An occurrence record for this genus had never been published from India, although images of these spiders from India have been on internet since 2010 (Kulkarni and Joseph 2015). No prior species information on this spider existed on IBP. Communication between the observer and the expert, through the portal, led to a collaborative effort in collecting specimens and verifying the identification. Having confirmed the species identification, the record was subsequently published as the first of genus *Siler* for India (Kulkarni and Joseph 2015). A species page was created on the portal for this spider species and descriptive content was populated in it by the expert. The publication was uploaded into the document module on the portal, leading to automated reciprocal linking from the species page to the document. A citizen science outreach initiative to aggregate more observations of this species resulted in over 15 records validated by experts (Fig. 3C). Images from observations uploaded by the general public have been extracted into the content of the species pages by curators. The species page automatically plots observations in its occurrence map (Fig. 3B). The still low number of observations does not allow a robust interpretation of the spatio-temporal distribution of that species but preliminary observations suggest a restricted spatial distribution within the Western Ghats and a peak in the monsoon season (July, Fig. 3D). Further observations will be needed to confirm these patterns. All records are available for download from the portal and the species page content is available for all to access. Finally, the species has now been included within the cleanlist of spider species on the IBP namelist module to reflect its curator-validated occurrence status in the country. This instance, which involved citizen participation, user-expert interaction, validation, information aggregation and open access availability of this information suitably demonstrates the utility of the portal in acting as an interaction medium between experts and enthusiastic citizens and as an information gathering and dissemination platform on species data.

**Discussion**

The India Biodiversity Portal facilitates the flow of biodiversity information between amateurs and experts by creating positive synergy between the civil society and the scientific community. It is successfully building relationships among a wide spectrum of stakeholders, such as research institutions, government or non-government organisations and amateur naturalists, between whom there is usually not many opportunities for communication. As a consequence, IBP is in line with the Biological Diversity Act 2002 (No.18 of 2003) of India, which followed the Convention on Biological Diversity in 1992, and argues that effective conservation of India’s biological resources, and its associated knowledge, should be done through an easy and sustainable access to biodiversity information (Gadgil 2003). IBP distinguishes itself from other initiatives in that it acts as a “one-stop shop” in its ability to aggregate various types of biodiversity data as well as provide a platform for collaboration upon it. IBP's approach of interconnected modules has two main advantages. First, it helps improve data quality, the responsibility of which remains with the community. Secondly, it allows the linking of biological data with other data, such as environmental (soil, climate) or social (population, administrative units) data and thus has the potential to evolve as a data-driven decision support system for biodiversity conservation and management. The availability of a wealth of spatial and temporal species distribution data and the portal’s ability to continuously aggregate such data through crowd-sourced participation make it an attractive platform to stakeholders. In a few years, IBP has accumulated a large amount of information on biodiversity, with an exponential increase in data, which has been already cited in academic research (36 publications have cited or used IBP data). We believe that in the next few years, data from IBP will considerably help our ability to understand the long (e.g. biogeographic history) and short (e.g. annual variation) term dynamics of Indian biodiversity, notably in a global change context.

Since open data is at the core of IBP’s philosophy, data will be shared with global initiatives such as the EoL and GBIF at regular intervals. Thus IBP, well illustrates how national initiatives working in a participatory manner and involving multi-institutional collaboration, may contribute to global biodiversity initiatives through a bottom-up perspective. Such bottom-up approaches may be easily replicated in other regions/countries as the code-base of the India Biodiversity Portal is available as open-source and is easily customisable. For instance, the core structure of IBP has already been used to set up a biodiversity platform for Bhutan (Bhutan Biodiversity Portal) and for weed documentation in the Western Indian Ocean (WIKWIO). Proliferation of such collaborative initiatives under open access paradigm facilitated by open source technology platform will strengthen biodiversity informatics as a discipline and also contribute towards enriching the collective knowledge of biodiversity and its effective conservation.

## Web location (URIs)

Homepage: http://indiabiodiversity.org

Download page: https://github.com/strandls/biodiv

Blog: http://blog.indiabiodiversity.org

## Technical specification

Platform: Grails

Programming language: Java and Groovy﻿

Operational system: Linux OS﻿

Interface language: Javascript, CSS, HTML

Service endpoint: http://indiabiodiversity.org

## Repository

Type: Git

Location: https://github.com/strandls/biodiv

## Usage rights

### Use license

Creative Commons Public Domain Waiver (CC-Zero)

## Figures and Tables

**Figure 6.**
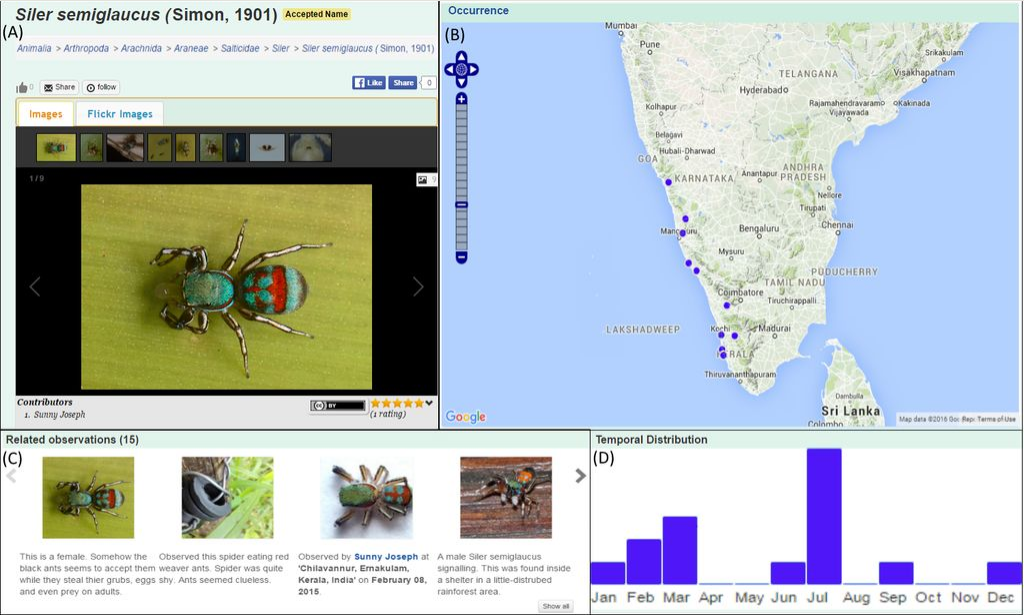
*Siler semiglaucus* species information aggregated by the portal. A composite image of screenshots from the portal showing (A) The species page gallery of *S. semiglaucus* with images extracted from observations uploaded by the public, (B) the distribution map within the species page, plotting all known occurrences uploaded as observations, (C) related observations previewing and linking to all observations of this species and (D) the temporal distribution of observations uploaded of this species.

## Footnotes

*1 Creative Commons (CC) license is a public copyright license that enables free distribution of an otherwise copyrighted work. A CC license protects people who use or redistribute an author's work from copyright infringement concerns as long as they abide by the conditions that are specified in the license by which the author distributes the work. There are several types of CC licenses, of which CC BY, CC BY-SA, and CC0 are considered conformant with Open content and data.
